# Seasonal Expression of NGF and Its Cognate Receptors in the Ovaries of Grey Squirrels (*Sciurus carolinensis*)

**DOI:** 10.3390/ani10091558

**Published:** 2020-09-02

**Authors:** Margherita Maranesi, Francesco Alessandro Palermo, Antonello Bufalari, Francesca Mercati, Daniele Paoloni, Paolo Cocci, Giulia Moretti, Silvia Crotti, Massimo Zerani, Cecilia Dall’Aglio

**Affiliations:** 1 Department of Veterinary Medicine, University of Perugia, via San Costanzo 4, 06126 Perugia PG, Italy; margherita.maranesi@unipg.it (M.M.); giuliamoretti89@gmail.com (G.M.); massimo.zerani@unipg.it (M.Z.); cecilia.dallaglio@unipg.it (C.D.); 2 School of Bioscience and Veterinary Medicine, University of Camerino, Via Gentile III Da Varano, I-62032 Camerino MC, Italy; francesco.palermo@unicam.it (F.A.P.); paolo.cocci@unicam.it (P.C.); 3 OIKOS Institute, Via Crescenzago 1, 20134 Milano MI, Italy; daniele.paoloni@istituto-oikos.org; 4 Experimental Zooprophylactic Institute of Umbria and Marche “Togo Rosati”, via Gaetano Salvemini, 1, 06126 Perugia PG, Italy; s.crotti@izsum.it

**Keywords:** nerve growth factor, neurotrophic tyrosine kinase receptor 1, nerve growth factor receptor, alien invasive species, reproduction, immunohistochemistry, real-time PCR, ELISA

## Abstract

**Simple Summary:**

Invasive alien species pose a significant threat to biodiversity, as once they have adapted to their new environment, they cause the reduction and even extinction of native species. In this framework, the American grey squirrel (*Sciurus carolinensis*) poses a serious threat to the European red species squirrel (*Sciurus vulgaris*), especially in the Umbria region of Italy. In fact, an invasive grey squirrel population has adapted well to the Umbrian territory, showing high reproductive success. In addition to its role in the development of the vertebrate nervous system, nerve growth factor (NGF) has recently been found to play an important role in reproduction. In order to investigate the reproductive physiology of female grey squirrels, the ovarian presence, distribution, and gene expression of NGF and its cognate receptors were evaluated during both breeding and nonbreeding seasons. The presence and gene expression of this system at the ovarian level, mainly during the breeding season, confirm the possible involvement of NGF and its receptors in the gonadal activity of this invasive grey squirrel population.

**Abstract:**

The grey squirrel is an invasive alien species that seriously threatens the conservation of the native red squirrel species. With the aim of characterizing the reproductive physiology of this species due to its great reproductive success, the function of the ovarian nerve growth factor (NGF) system was analyzed in a grey squirrel population living in central Italy. During the breeding and nonbreeding seasons, the ovarian presence, distribution, and gene expression of NGF, neurotrophic tyrosine kinase receptor 1 (NTRK1), and nerve growth factor receptor (NGFR), as well as NGF plasma concentrations, were evaluated in female grey squirrels. NGF was found in the luteal cells and in the thecal and granulosa cells of follicles, while NTRK1 and NGFR were only observed in follicular thecal and granulosa cells. *NGF* and *NGFR* transcripts were almost two-fold greater during the breeding season, while no seasonal differences were observed in *NTRK1* gene expression. During the breeding season, NGFR was more expressed than NTRK1. Moreover, no changes were observed in NGF plasma levels during the reproductive cycle. The NGF system seems to be involved in regulating the ovarian cycle mainly via local modulation of NGF/NGFR, thus playing a role in the reproductive physiology of this grey squirrel population.

## 1. Introduction

Nerve growth factor (NGF) was the first member of the neurotrophin family [1] to be discovered, which was followed by the brain-derived growth factor (BDNF) and other neurotrophins. NGF exerts its biological action through two receptors, both belonging to the tumor necrosis factor receptor family [2]: the high-affinity neurotrophic tyrosine kinase receptor 1 (NTRK1, 140-kDa) and the low-affinity nerve growth factor receptor (NGFR, 75-kDa), previously known as tropomyosin receptor kinase A and p75, respectively. After its discovery, NGF proved to be essential for nervous system development and the differentiation and survival of neurons [3,4]; recent studies have shown that NGF can enhance male and female reproductive functions [5,6]. In females, the expression of this neurotrophin varies significantly during the phases of the ovarian cycle and pregnancy due to hormonal changes [7]. More specifically, NGF expression and its receptors were evaluated in the uterus of several mammalian species such as rat, guinea pig, mouse, bat, pig, horse, sheep, and rabbit [8,9,10,11,12] and also in the ovaries of mice, rats, hamsters, sheep, Shiba goats, bovines, and rabbits [13,14,15,16,17,18,19,20,21], thus highlighting that the NGF system plays an essential autocrine and/or paracrine role in the development of oocytes and follicles [22]. Li et al. [22] recently found the NGF system expressed in the uterus of a wild female ground squirrel, while Bao et al. [23] found the NGF system expressed in the ovarian surface epithelium.

The eastern grey squirrel (*Sciurus carolinensis*) belongs to the order of Rodentia, family Sciuridae, and is native to eastern North America; it is a medium-sized tree squirrel in the genus *Sciurus* with no gender differences in size and coat color between males and females [24]. The grey squirrel is considered to be one of the worst pest species in Europe and worldwide; in fact, it has been included among the world’s 100 worst invasive alien species (IAS) by the International Union for the Conservation of Nature [25].

The European Union strongly opposes the introduction of IAS and has recently implemented Regulation n. 1143/2014 on the prevention and management of the introduction and spread of IAS.

In this context, a classic example in Europe of the competitive exclusion of a native species by an alien species is the replacement of the native red squirrel (*Sciurus vulgaris*) by the introduced grey squirrel in the United Kingdom and Italy [26,27,28]. In our recent study [29], it was observed that the grey squirrel females belonging to the Umbrian grey squirrel population (central Italy) have two mating seasons, one from December to February and the other from April to June, with two peaks of litter production [21,24,30]. This seasonal breeding activity is interrupted by anestrous phases that generally occur in March and from July to November [29].

Various authors have highlighted the importance of gaining a better understanding of the reproductive system of this invasive species since it adapts very well to new environments and is characterized by a greater reproductive success than the native species [29,31,32]. Therefore, the aim of this study is to investigate the ovarian presence, distribution, and gene expression of NGF, NTRK1, and NGFR, as well as plasma NGF levels, during both breeding and nonbreeding seasons.

## 2. Materials and Methods

### 2.1. Animal Capture and Sample Collection

As part of the LIFE U-SAVEREDS Project (LIFE13 BIO/IT/000204) “Management of grey squirrel in Umbria: conservation of red squirrel and preventing loss of biodiversity in Apennines” investigating the conservation of the European red squirrel and forest ecosystem in Umbria and central Italy [33], during the 2016–2018 control campaign, female grey squirrels were captured using Tomahawk live-traps (mod. 202.5, Tomahawk Live Trap Co., Hazelhurst, WI, USA) in both breeding (n = 5) and nonbreeding (n = 5) seasons. The traps were sheltered against sunlight and set far from busy roads in order to minimize stress in the captured squirrels [29]. The squirrels were captured in compliance with regulations regarding wildlife control laid down in Art. 19 of the Italian Law 157/92 “Rules for the protection of wild animals and homeotherms and for hunting”, Habitat Directive 92/43/CEE, and Regulation n. 1143/2014 (22 October 2014) of the European Parliament (on the prevention and management of the introduction and spread of invasive alien species). The trapped animals were surgically gonadectomized [29,34], which allowed the collection of their ovaries. Within a few minutes, the ovaries to be used for investigating the gene expression were washed in an RNase-free phosphate-buffered saline solution and then frozen at −80 °C [35], while the ovaries that were to undergo immunohistochemical evaluation were fixed in 4% (*w*/*v*) formaldehyde in PBS (pH 7.4) for 24 h at room temperature and then processed using routine tissue processing techniques [36]. Blood samples (1 mL) were taken from the radial vein of each anesthetized squirrel just before surgery. Plasma was obtained by centrifuging blood samples (1 mL) at 1500× *g* for 10 min. The plasma was stored at −20 °C and successively tested for NGF.

### 2.2. Immunohistochemistry

The immunohistochemical procedure was carried out as previously described [37]. The tissue samples from each squirrel were embedded in paraffin wax and serially sectioned at 4–5 mm. The sections were placed on poly-L-lysine-coated slides, deparaffinized, rehydrated through graded ethanol to distilled water, and then exposed to microwave irradiation for 15 min at 750 W in citrate buffer solution (10 mM, pH 6) for antigen retrieval. After they had cooled to room temperature, the slides were rinsed with phosphate-buffered saline solution (PBS). All of the subsequent steps were carried out in a moist chamber at room temperature to prevent the evaporation of the reagents. The endogenous peroxidase activity of the tissue sections was quenched by incubating the slides in a peroxidase blocking solution (3% H2O2 in PBS) for 10 min. Nonspecific binding was blocked by incubating the sections in normal horse serum (diluted 1:10 for 30 min, S-2000, from Vector Laboratories, Burlingame, CA, USA). The serial sections were then incubated in the following primary monoclonal antisera—mouse anti-NGF (sc-365944), mouse anti-NTRK1 (sc-803998), and mouse anti-NGFR (sc-271708)—which were all purchased from Santa Cruz Biotechnology (Santa Cruz, CA, USA), diluted 1:100 in PBS, and incubated overnight. After incubation, the slides were rinsed 3 times with PBS buffer (5 min each) and incubated with biotinylated horse anti-mouse IgG secondary antibody (diluted 1:200 in PBS for 30 min, BA-2000, from Vector Laboratories). After washing with PBS, the slides were exposed to the avidin–biotin complex for 30 min (ABC Kit, PK-4000, from Vector Laboratories) and then rinsed again with PBS. The peroxidase activity sites were visualized using 3,3′diaminobenzidine-4-HCL as chromogen (DAB, SK-4100, from Vector Laboratories); the slides were washed in distilled water, counterstained with Mayer’s hematoxylin, rinsed under running tap water and mounted with Eukitt medium (O3989 from Sigma-Aldrich, Alcobendas, Spain) for light microscopy. Positive reactions were indicated by reddish-brown staining. Control sections of nonspecific staining were prepared by omitting the primary antibodies and incubating the slides with PBS or mouse immunoglobulin G (IgG). Three independent observers, who were unaware of the period analyzed, evaluated five microscopic fields for both the breeding and nonbreeding seasons, and staining intensity was expressed in arbitrary units as follows: negative (−), weak (±), moderate (+), strong (++).

### 2.3. RNA Extraction and Real-Time PCR

Total RNA was extracted from the ovaries, as previously reported [38]. Five µg of total RNA was reverse-transcribed in 20 µL of iSCRIPT cDNA using random hexamer according to vendor protocol (Bio-Rad Laboratories, Milan, Italy). Genomic DNA (gDNA) contamination was verified by developing a PCR without reverse transcriptase. Serial experiments were carried out to optimize the quantitative reaction, efficiency, and Ct values. The optimal 25 µL PCR reaction volume contained 12.5 µL of iQSYBR Green SuperMix (Bio-Rad Laboratories), 1 µL forward and reverse primers (stock concentration of 10 µM), and water to 25 µL. The primers used in this study are listed in Table 1.

All of the reagents were mixed as a master mix and placed into a 96-well PCR plate before adding 2 µL of cDNA (10-fold diluted with water). The amplification fidelity of the samples was also verified using agarose gel electrophoresis for two animals belonging to different groups. The images of gels were acquired using a Kodak DC290 digital camera. PCR was performed using an iCycleriQ (Bio-Rad Laboratories) with an initial incubation at 95 °C for 1.5 min, followed by 40 cycles at 95 °C for 15 s, 53 °C for 30 s, during which fluorescence data were collected. The threshold cycle (Ct value) was set automatically for each trace.

Standard curves were generated by plotting the threshold value (Ct) against the log cDNA standard dilution (1/10 dilution) in nuclease-free water. The slope of these graphs was used to determine the reaction efficiency. Immediately after PCR, the melting curve analysis was carried out in order to determine the specificity of each primer set. A melting curve protocol was performed by repeating 80 heating cycles for 10 s, from 55 °C with 0.5 °C increments, during which fluorescence data were collected. The analysis demonstrated that a single product was generated for each reaction, thus confirming the specificity of the primer pairs. Sample mRNA quantification was evaluated using iCycler system software, while mRNA gene expression was quantified using the 2^−ΔΔCt^ method [39,40]. The beta-actin Ct housekeeping gene (*BACT*) was determined in order to normalize sample variations in the amount of starting cDNA. 

### 2.4. NGF Plasma Levels

The enzyme-linked immunosorbent assay (ELISA) was utilized to determine plasma NGF concentrations using the DuoSet ELISA for measuring NGF (R&D System, Milan, Italy), according to the manufacturer’s instructions [41].

### 2.5. Statistical Analysis

Welch’s ANOVA/Welch’s *t*-test were used to analyze all data. Differences were considered significant at *p* < 0.05.

## 3. Results

### 3.1. Immunohistochemistry

**Figure 1:**
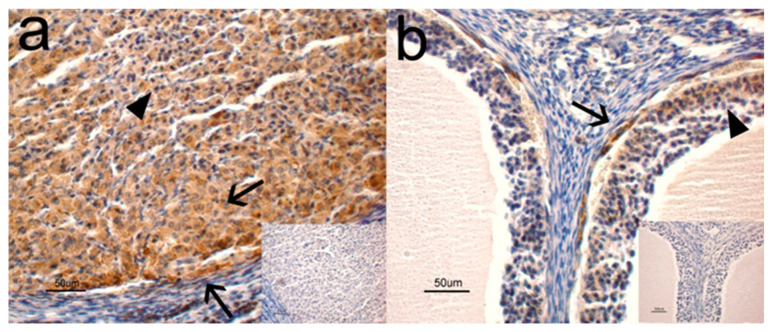
Nerve growth factor (NGF) immunoreaction (IR) in the ovaries of grey squirrels. (**a**) The positive signal is localized in the cytoplasm of small (arrowhead) and large (arrows) luteal cells. (**b**) The positive signal is localized in the cytoplasm of thecal (arrow) and granulose (arrowhead) cells. Bar = 50 μm.

**Figure 2:**
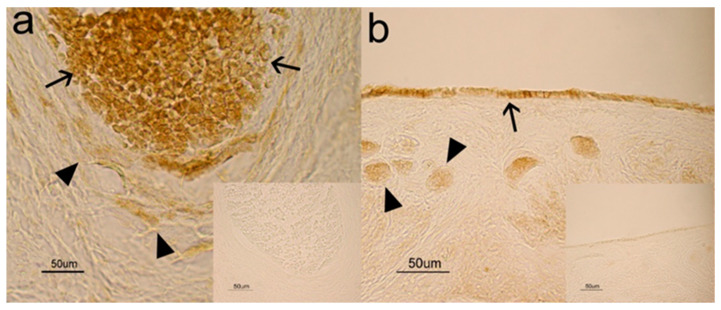
Nerve growth factor receptor (NGFR) (**a**) and neurotrophic tyrosine kinase receptor 1 (NTRK1) (**b**) IR in the ovaries of grey squirrels. In both cases, the positive reaction is localized in the cytoplasm of thecal (arrowheads) and granulose (arrows) cells (**a**) and in germinative epithelium (arrow) and follicular cells (arrowheads). Bar = 50 μm.

The immunohistochemical analysis showed a positive immunoreaction (IR) for NGF and its cognate receptors, NTRK1 and NGFR, in the ovaries. More specifically, NGF was observed in the cytoplasm of small (Figure 1a, arrowhead) and large (Figure 1a, arrows) luteal cells and in the cytoplasm of thecal cells (Figure 1b, arrow) and granulose cells (Figure 1b, arrowhead). NTRK1 and NGFR were found in both thecal (Figure 2a, arrowheads) and granulose (Figure 2a, arrows) cells and in the cytoplasm and perinuclear localization of follicles at different stages of maturation. For both receptors, no positive IR was found in the luteal cells. Staining was never observed in the control sections where the primary antibody was omitted (Figure 1 and Figure 2, inserts).

Ovarian structures and cells showed strong NGF and NGFR staining intensity and moderate NTRK1 staining intensity. No differences were observed between the different time periods analyzed (Table 2).

Intensity of staining in tissue sections was scored as follows: negative (−), weak (±), moderate (+), strong (++). No differences were observed between breeding and nonbreeding animals.

### 3.2. Real-Time PCR

**Figure 3:**
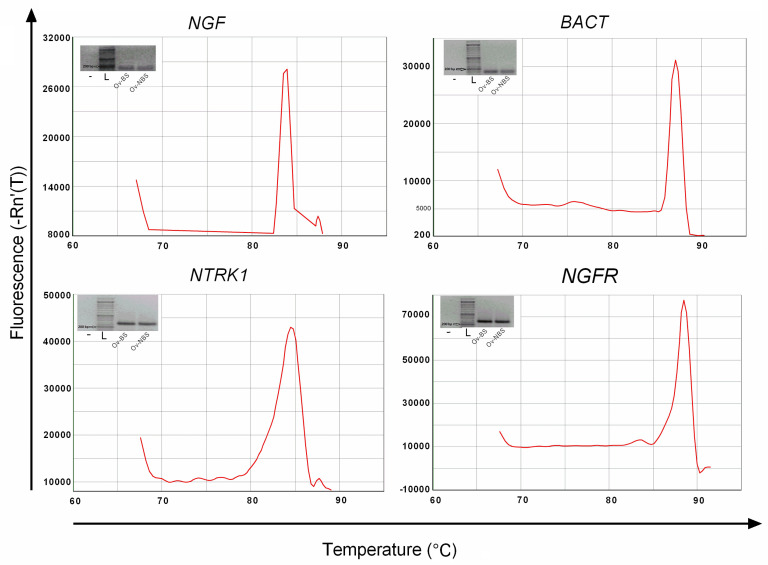
Specificity of primers and amplicon length. Both the presence of a single band with the expected size in 1.2% agarose gel electrophoresis and the single peak of dissociation curves indicate that the PCR products were specific. Bp: base pairs; −: negative control; L: ladder; Ov-BS: ovary—breeding season; Ov-NBS: ovary—nonbreeding season; *NGF*: nerve growth factor, *NTRK1*: neurotrophic tyrosine kinase receptor 1; *NGFR*: nerve growth factor receptor target genes; *BACT*: β-actin housekeeping gene.

Each pair of primers used in this study showed good specificity and sensitivity when a single-fragment often-expected fragment size was amplified (Figure 3). This estimation indicated that the cross-species primers can correctly amplify the identified target genes in the grey squirrel.

**Figure 4:**
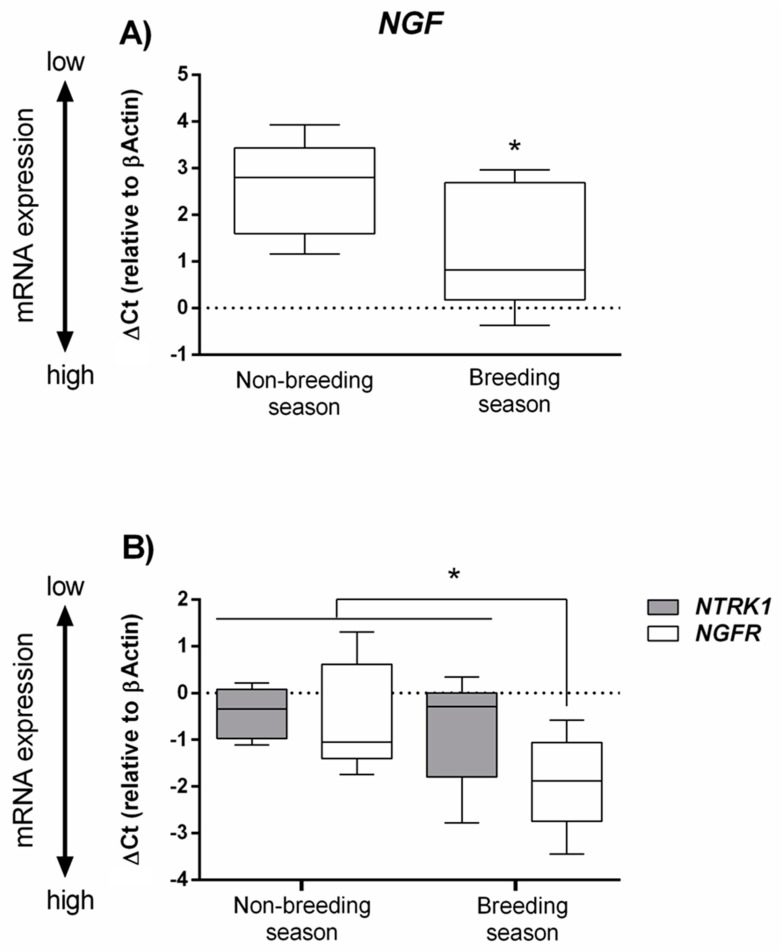
Box-and-whisker plots of nerve growth factor (*NGF*; panel **A**) and neurotrophic tyrosine kinase receptor 1 (*NTRK1*) and nerve growth factor receptor (*NGFR*) (panel **B**) transcription values (medians and 95% confidence intervals) in ovaries of grey squirrels during breeding (January–February) and nonbreeding (July–November) seasons. Data are shown as ΔCt (relative to β-actin) values, with higher ΔCts representing lower expression (Welch’s ANOVA/Welch’s *t*-test: * *p* < 0.05).

The mRNA expression level of *NGF* and its receptor *NGFR* was significantly upregulated during the breeding season compared with the nonbreeding season (Figure 4). Contrastingly, no differences were observed in the *NTRK1* gene expression between the two breeding seasons (Figure 4). *NGFR* was more expressed than *NTRK1* during the breeding season, while no differences between these two receptors were observed during the nonbreeding season (Figure 4).

### 3.3. NGF Plasma Levels

Plasma NGF concentrations did not change significantly during the reproductive cycle (nonbreeding season: 78 ± 15 pg/mL; breeding season: 85 ± 12 pg/mL).

## 4. Discussion

An extensive bibliography confirms the role of NGF in the ovarian function of mammals [13,42,43,44]. However, to the best of our knowledge, this is the first study evaluating the NGF system in female grey squirrels during the different phases of the reproductive cycle.

This study highlights the presence of NGF and its receptors in the ovaries of grey squirrels as positive immunosignals were detected in the luteal and follicular cells, demonstrating characteristic cytoplasmic reactivity. The presence of NGF in the ovaries confirms that it is commonly found in the ovaries of mammalian species [13,14,15,16,17,18,19,20,21,23] and requires the definition of a potential function of this molecule in controlling grey squirrel reproductive activity.

The gene and protein expression of NGF, NTRK1, and NGFR detected in the ovaries during the mating season and in the anestrous phase indicates the possible synthesis input of NGF and its receptors and the secretion of this neurotrophin in the ovaries of the grey squirrel. This is in agreement with the results obtained by other authors [19,21], where *NGF*, *NTRK1*, and *NGFR* mRNA and protein were described in some rabbit ovarian tissues, especially in granulosa and thecal cells, [21] as well as in bovine thecal cells [19].

Interestingly, the gene expression levels of *NGF* and its low-affinity receptor *NGFR* showed significantly higher differences in the breeding season than the nonbreeding season, whereas *NTRK1* mRNA levels did not change in the two reproductive periods examined. Contrastingly, no significant difference was observed in the protein staining for NGF and its receptors. The differences in the expression of the NGF system transcript and protein detected may be due to differences in the sensitivity of the techniques used (real-time PCR vs. IHC) or to the activation of post-transcriptional mechanisms, resulting in the failure to translate all of the transcripts into protein [45]. Further studies are required in order to gain a better understanding of these mechanisms. Although NGFR is considered an auxiliary receptor of NTRK1, which plays a modulatory role in NGF biological action through the activation of the NTRK1 receptor [46], the data obtained suggest that this low-affinity receptor may play a more important role than high-affinity NTRK1 in NGF-regulated ovarian function in grey squirrels. The *NGF* and *NGFR* gene expression in ovaries increased during the breeding season, accompanied by a distinct morphological modification, such as the formation of large tertiary follicles [29]. This finding suggests that besides its ability as a growth factor in the nerve structures of the developing follicles, NGF could play an important role in competence acquisition in the steroidogenesis of follicular cells in this squirrel species [47].

Our results partially differ from the data obtained by Li et al. [48] during a study focused on the ovaries of wild ground squirrels, in which higher levels of *NGF*, *NTRK1,* and *NGFR* transcripts and proteins were observed in the breeding season. These authors hypothesized that NGF and its cognate receptors’ expression levels are modulated by gonadotropins and/or by locally produced hormones [48]. The same authors have made similar considerations in the uterus under the same experimental conditions during both the reproductive and nonreproductive seasons [22].

The higher levels of NGF transcripts observed during the breeding season suggest another NGF-induced action on the grey squirrel ovary, as reported for llama by Valderrama et al. [47]. These authors found that the systemic administration of extracted and purified NGF from llama seminal fluid induced a rapid shift from estradiol to progesterone production in the preovulatory follicle, probably due to an NGF-induced difference in gene expression patterns of follicular steroidogenic enzymes [47]. In llamas and alpacas, the administration of NGF to females induces ovulation [49]. In rabbits, NGF is mainly synthesized by the uterine wall following semen contact and acts on the ovaries and uterine/cervix afferent neurons projecting LH surge hypothalamic centers, thus inducing ovulation [21]. It is difficult to accurately pinpoint the moment of ovulation in a free-living animal species like the squirrel, which cannot be kept in captivity; however, the numerous studies carried out on other species [16,43] suggest that NGF plays an important role in periovulatory processes that could also be decisive in the squirrel.

In 1999, Mattioli et al. [16] demonstrated the in vitro production of NGF by medium–large ovine follicles stimulated by gonadotropins and related it to ovarian functional status [16]. Although gonadotropin plasmatic levels or their local production were not analyzed during the two periods, it is possible that gonadotropins may affect ovarian NGF production in squirrels.

In this study, only NGF, without its cognate receptors, was detected in luteal cells, suggesting that NGF plays an indirect role in the regulation of the corpus luteum lifespan. In this context, using NGF purified from llama seminal plasma, Silva et al. [50] observed an increase of vascularization in the llama corpus luteum, the upregulation of *CYP11A1/P450scc* and *STAR* transcripts, and an increase in progesterone secretion, suggesting that NGF has a luteotrophic effect. Similar results were obtained in heifers by both Tribulo et al. [51] and Stewart et al. [52]; bovine seminal plasma was used in the first study, which considerably increased ovulation synchrony and progesterone production after ovulation [51]; in the second study, purified NGF administered at the time of artificial insemination increased stimulated progesterone production by the corpus luteum and conceptus development [52]. The luteotrophic effect of NGF in llamas and alpacas has also been reported [53,54,55].

To date, no studies have been carried out on the plasma levels of NGF in grey squirrels. From our studies, these plasmatic values, which ranged from 78 pg/mL in the nonbreeding season to 85 pg/mL in the breeding season, were higher than the plasma levels observed in nulliparous mice (~10 pg/mL) [56] and rabbit does (50–60 pg/mL) [21] and lower than in adult rat females (40 ng/mL) [57], dogs (15–35 ng/mL) [58], and humans (200 pg/mL) [59].

## 5. Conclusions

In conclusion, our results suggest a possible involvement of the NGF system in grey squirrel seasonal reproductive activity, via either auto- and/or paracrine mechanisms, as previously demonstrated in other mammalian species [22]. Further research should evaluate the role of NGF and its cognate receptors in the reproductive mechanisms of the IAS grey squirrel, focusing on the biological functions of NGFR and the NGF source.

## Figures and Tables

**Table 1:** Primers for *NGF*, *NTRK1*, *NGFR,* and *BACT* (used as internal standard) for real-time PCR quantification.

Gene		Primers	bp
*NGF*	F	TCCACCCACCCAGTCTTC	178
	R	GCTCGGCACTTGGTCTCA	
*NTRK1*	F	TCGGACCATGCTGCCCATCC	261
	R	AGGCGTTGCTGCGGTTCTCG	
*NGFR*	F	GGAGGACACGAGTCCTGAGC	295
	R	CAGTGGAGAGTGCTGCAAAG	
*BACT*	F	TTGTGATGGACTCCGGAGAC	186
	R	TGATGTCACGCACGATTTCC	

*NGF*: nerve growth factor; *NTRK1*: neurotrophic tyrosine kinase receptor 1; *NGFR*: nerve growth factor receptor; *BACT*: β-actin; bp: base pair.

**Table 2:** Immunostaining intensity.

	NGF	NTRK1	NGFR
Breeding	++	+	++
Nonbreeding	++	+	++
